# Comparison of ^68^Ga-FAP-2286 and ^18^F-FDG PET/CT in the diagnosis of advanced lung cancer

**DOI:** 10.3389/fonc.2024.1413771

**Published:** 2024-07-01

**Authors:** Feifan Xiang, Yue Zhang, Xiaoqi Tan, Jintao Zhang, Tengfei Li, Yuanzhuo Yan, Wenzhe Ma, Yue Chen

**Affiliations:** ^1^ The State Key Laboratory of Quality Research in Chinese Medicine, Macau University of Science and Technology, Macau, Macau SAR, China; ^2^ Department of Orthopedic, the Affiliated Hospital, Southwest Medical University, Luzhou, China; ^3^ Department of Nuclear Medicine, the Affiliated Hospital, Southwest Medical University, Luzhou, China; ^4^ Department of Dermatology, the Affiliated Hospital, Southwest Medical University, Luzhou, China; ^5^ Nuclear Medicine and Molecular Imaging Key Laboratory of Sichuan Province, Luzhou, China; ^6^ Institute of Nuclear Medicine, Southwest Medical University, Luzhou, China

**Keywords:** Gallium-68, FAP-2286, PET/CT, lung cancer, metastasis detection

## Abstract

**Purpose:**

The ^68^Ga/^177^Lu-FAP-2286 is a newly developed tumor imaging agent that shows potential for visualizing and treating tumor stroma. The objective of this research was to evaluate the effectiveness of ^68^Ga-FAP-2286 PET/CT and ^18^F-FDG PET/CT in diagnosing advanced lung cancer.

**Methods:**

In this prospective study, patients with lung cancer who underwent ^68^Ga-FAP-2286 and ^18^F-FDG PET/CT examinations between September 2022 and June 2023 were analyzed. Lesion uptake was converted to SUVmax. A paired T-test was used to compare the SUVmax, and the number of positive lesions detected by the two methods was recorded.

**Results:**

In total, 31 participants (median age: 56 years) were assessed. The uptake of ^68^Ga-FAP-2286 was significantly higher than that of ^18^F-FDG in primary lesions (9.90 ± 5.61 vs. 6.09 ± 2.84, respectively, P < 0.001), lymph nodes (7.95 ± 2.75 vs. 5.55 ± 1.59, respectively, P=0.01), and bone metastases (7.74 ± 3.72 vs. 5.66 ± 3.55, respectively, P=0.04). Furthermore, the detection sensitivity of lymph nodes using ^68^Ga-FAP-2286 PET/CT was superior to that with ^18^F-FDG PET/CT [100% (137/137) vs. 78.8% (108/137), respectively], as well as for bone metastases [100% (384/384) vs. 68.5% (263/384), respectively]. However, the detection sensitivity for primary tumors using both modalities was comparable [100% (13/13) for both].

**Conclusion:**

Compared to ^18^F-FDG PET/CT, ^68^Ga-FAP-2286 PET/CT demonstrated better lesion detection capabilities for lung cancer, particularly in lymph nodes and bone metastases, providing compelling imaging evidence for the efficacy of ^177^Lu-FAP-2286 treatment.

## 1 Introduction

The incidence of lung cancer is increasing annually, making it the second most common malignant tumor (1). Most of the patients are diagnosed at advanced stages. Early prevention, diagnosis, and treatment of lung cancer remain a great challenge for humankind (2). In recent years, molecular imaging of lung cancer has revolutionized the diagnosis and treatment of lung cancer, and accurate radiological evaluation of specific tumor stroma is crucial (3).

Positron emission tomography (PET)/computed tomography (CT) is increasingly gaining popularity for detecting and evaluating malignant tumors owing to its targeted and non-invasive nature. In particular, ^18^F-FDG PET/CT has demonstrated superiority over traditional diagnostic methods in lung cancer staging, earning recognition in international guidelines as a preferred screening tool (4, 5). Nevertheless, a drawback of false positives arising from FDG uptake in inflammatory lesions poses a challenge (6). Furthermore, the diagnostic performance of ^18^F-FDG PET/CT for detecting bone metastases and occult pleural metastases is suboptimal (7). Studies have also highlighted the limitation of high ^18^F-FDG uptake in reactive lymph nodes in the mediastinum and bilateral hilar regions, complicating the diagnosis of malignant stages (8, 9). Therefore, there is a pressing need to develop new imaging tracers to address these challenges.

Fibroblast-activating protein (FAP) is expressed in more than 90% of epithelial cancers, while its presence in normal adult tissues is notably low (10). Given their limited expression profile and function, ^68^Ga-labeled FAP inhibitor variants, including FAPI-04/46/74, are considered novel broad-spectrum tumor imaging agents with broad application prospects (10). However, their relatively short retention time in tumors may limit their applicability in radionuclide therapy (11, 12).

Distinguished by its characteristics as a low-molecular-weight polypeptide with a cyclic peptide-binding motif, FAP-2286 exhibits high selectivity for FAP, stability in human plasma, and prolonged retention time in tumors (13). Particularly in cancer types demonstrating low to moderate uptake of ^18^F-FDG, FAP-2286 emerges as a promising alternative to ^18^F-FDG (14). Preclinical studies have shown that the biological distribution, dosimetry, and tumor uptake of ^68^Ga-FAP-2286 are similar to previously reported FAPI compounds. Additionally, the uptake of ^68^Ga-FAP-2286 was consistently higher than that of ^18^F-FDG. These results are particularly relevant in differentiating tumors from inflammatory uptake and small-volume diseases (15). Despite these advantages, clinical reports on the application of ^68^Ga-FAP-2286 in lung cancer remain limited. Most reported studies have compared ^68^Ga-FAP-2286 with ^18^F-FDG in patients with a various cancers, but the number of included cases is small, typically ranging from a few to a dozen. The largest study included 64 patients with 15 different cancers, but it involved only two cases of lung cancer. Consequently, the diagnostic value of ^68^Ga-FAP-2286 in lung cancer may be underexplored (14, 16–18).

Therefore, this study focused on assessing the utility of ^68^Ga-FAP-2286 PET/CT for evaluating primary or recurrent lung cancer tumors by including more patients with lung cancer. The hypothesis was that it could serve as a viable alternative to ^18^F-FDG PET/CT, with the potential for integrating ^68^Ga/^177^Lu-FAP-2286 in diagnosing and treating lung cancer. In this report, we present the outcomes of a prospective study comparing the effectiveness of ^68^Ga-FAP-2286 PET/CT against ^18^F-FDG PET/CT in detecting lung cancer.

## 2 Methods

### 2.1 Study participants

This single-center prospective study was conducted at the Affiliated Hospital of Southwest Medical University. The study was approved by the Institutional Review Board, registered on the Chinese Clinical Trial Registry website (http://chictr.org.cn, ChiCTR2100044131), and obtained ethical clearance from the Ethics Committee (AHSWMU-2020-035). All participants provided written informed consent before receiving treatment. Patients were continuously enrolled at the Affiliated Hospital of Southwest Medical University from September 2022 to June 2023, with ^68^Ga-FAP-2286 PET/CT conducted after ^18^F-FDG PET/CT to facilitate a comparative analysis without affecting patient care.

The inclusion criteria were (a) newly diagnosed or previously treated lung cancer, (b) histologically confirmed lung cancer, and (c) consent to the above two PET/CT examinations. The exclusion criteria were as follows: (a) non-malignant diseases; (b) other primary malignancies at the time of examination; (c) severe hepatic and renal insufficiency; (d) pregnancy; (e) refusal to be scanned by FDG or FAP-2286; and (f) inability or unwillingness to provide written informed consent by the study participants, parents, or legal representatives.

The histopathological results were used to determine the final diagnosis of all primary or recurrent tumors. Due to technical and ethical limitations, it is not possible to histologically validate all lymph nodes and distant metastases. When histopathological findings are not available, the final diagnosis is determined by CT features and other imaging tests (magnetic resonance imaging, enhanced CT, ultrasound, bone scan), and corresponding follow-up observations. The follow-up period was not less than three months. At follow-up treatment (radionuclide therapy, radiotherapy, chemotherapy, targeted therapy, etc.), significant reduction in tumor size or progression was determined to be malignant.

### 2.2 Image acquisition

This study was conducted using a hybrid PET/CT scanner (uMI780; United Imaging Healthcare, Shanghai, China). The ^68^Ga-FAP-2286 PET/CT scan was conducted within 1 week following the ^18^F-FDG PET/CT scan. Intravenous doses of ^18^F-FDG and ^68^Ga-FAP-2286 were calculated based on the patient’s body weight (FDG 3.7 MBq/kg; FAP-2286 1.8–2.2 MBq/kg). Patients were asked to fast for at least 5 hours before ^18^F-FDG PET/CT, avoid strenuous activity or prolonged exercise, and ensure that their peripheral blood glucose levels were normal. No specific preparations (such as fasting and normal blood glucose levels) were necessary on the day of the ^68^Ga-FAP-2286 PET/CT examination. PET/CT imaging was performed 1 h after intravenous administration. The specific imaging process and instrument parameters were as reported previously (19). Upon completing the scanning process, the results were sent to the post-processing workstation for image reconstruction. Nuclear medicine physicians assessed the overall condition of each patient, including temperature, heart rate, blood pressure, and mental state, within 2 h of injection.

### 2.3 Image analysis

Visual, qualitative, and semi-quantitative interpretations of ^18^F-FDG and ^68^Ga-FAP-2286 PET/CT were independently performed by two experienced nuclear medicine physicians, each with at least 10 years of PET/CT imaging experience. Differences were resolved through discussion and consensus. The PET/CT images of the patients were evaluated in the coronal, axial, and sagittal planes. On visual assessment, any focal tracer accumulation of ^18^F-FDG and ^68^Ga-FAP-2286 higher than that in the adjacent tissue or background was considered a positive lesion or suspected malignant lesion. Positive lesions were combined with the corresponding CT scan image data for further diagnosis and classified as non-malignant lesions, primary tumors, distant metastases, or lymph node metastases, and the number of lesions was counted. In addition to visual assessment, a semi-quantitative assessment of lesions was performed by plotting the areas of interest along the lesion edges on axial PET images. SUVmax was automatically calculated using an advanced workstation to quantify tracer uptake in the lesions. The metabolic tumor volume of the primary tumor was measured on ^68^Ga-FAP-2286 and ^18^F-FDG PET/CT images using the threshold method (45.0% SUVmax).

### 2.4 Statistical analysis

All statistical analyses were conducted using SPSS software (version 26.0). Continuous data were expressed as mean ± standard deviation. The detection rate of ^68^Ga-FAP-2286 and ^18^F-FDG was compared by McNemar test. The difference between SUVmax was determined using the paired t-test. Statistical significance was set at P < 0.05.

## 3 Results

### 3.1 Participant characteristics

From September 2022 to June 2023, 31 patients (24 men and 7 women) were enrolled in this study, with a median age of 56 years (quartile range: 53–66 years). Among them, 13 patients were newly diagnosed with primary lung cancer, while 18 had recurrent or post-treatment metastatic lung cancer. Further, 24 participants presented with stage IV lung cancer. Most participants were diagnosed with non-small cell carcinoma (25 cases), followed by small cell carcinoma (6 cases). Based on their individual conditions, participants predominantly opted for chemotherapy (6 cases) or targeted therapy (7 cases) as treatment modalities (**Table 1**).

**Table 1:** Demographic and clinical features of the 31 participants with lung cancer.

Characteristics	Value
**No. of patients**	31
Age(y)	
Median	56
Interquartile range	53-66
Sex	
Men	24
Women	7
Histopathologic findings	
Non-small cell carcinoma	25
Small cell carcinoma	6
Indication for PET	
Initial assessment(staging)	13
Recurrence detection(restaging)	18
Staging	
II	1
III	6
IV	24
Patient status	
Treatment-naive	11
Resection surgery	2
Chemotherapy	6
Radiotherapy	2
Targeted therapy	7
Immunotherapy	2

Lung cancer staging was performed according to the 8th edition of the American Joint Committee on Cancer Staging Manual (20).

### 3.2 Detection of primary and recurrent patients

**Figure 1:**
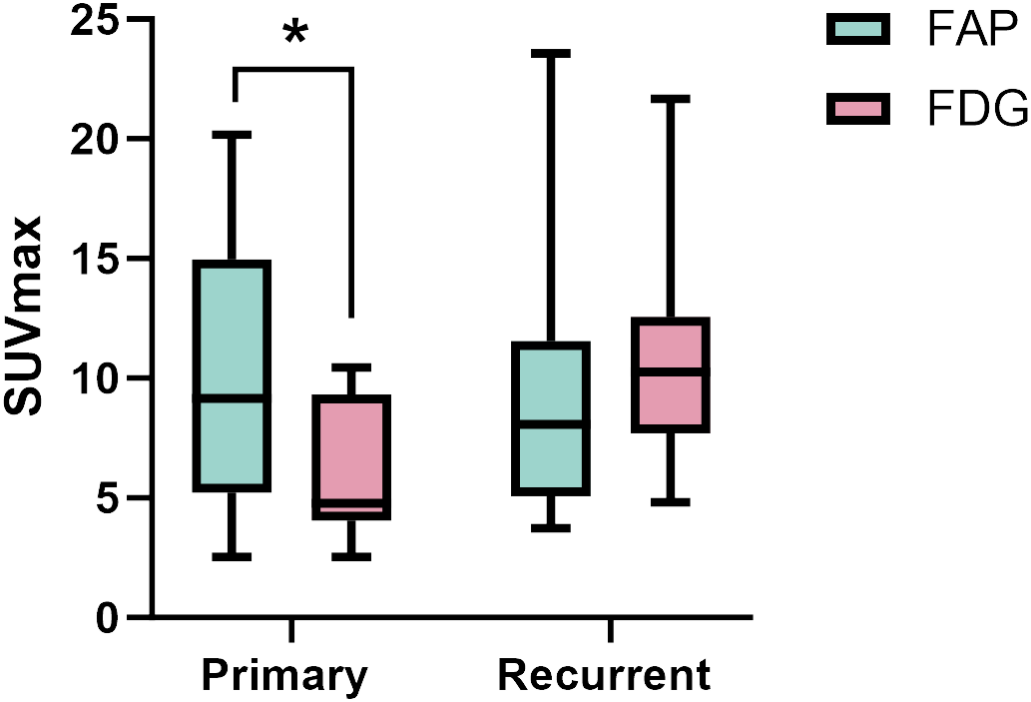
The SUVmax of FAP-2286 and FDG in primary and recurrent tumors. The accumulation of ^68^Ga-FAP-2286 was significantly higher than that of ^18^F-FDG in primary tumors (9.90 ± 5.61 vs. 6.09 ± 2.84, P < 0.01). There was no difference between ^68^Ga-FAP-2286 and ^18^F-FDG in recurrent tumors (9.44 ± 5.34 vs. 11.14 ± 4.80, P=0.28). * indicates P < 0.05.

For individual primary tumor analysis (n=13), the SUVmax of ^68^Ga-FAP-2286 was substantially higher than that of ^18^F-FDG PET/CT (9.90 ± 5.61 vs. 6.09 ± 2.84, respectively, P < 0.01). Moreover, the sensitivity for ^68^Ga-FAP-2286 PET/CT and ^18^F-FDG PET/CT was 100% (13/13) in detecting primary tumors. Similarly, in the case of recurrence detection after surgery among 18 patients, both modalities showed a sensitivity rate of 100% (18/18). There was no significant difference between the SUVmax of ^68^Ga-FAP-2286 and ^18^F-FDG (9.44 ± 5.34 vs. 11.14 ± 4.80, respectively, P=0.28). Detailed comparative results are presented in **Figure 1** and **Table 2**.

**Table 2:** Comparison of tracer uptake in the lesions between ^68^Ga-FAP-2286 and ^18^F-FDG PET/CT in participants with primary and recurrent lung cancer (n=31).

Tumor Lesions	^68^Ga-FAP-2286 PET/CT	^18^F-FDG PET/CT	P Value
Primary tumors			
No. of lesions	13	13	
Mean SUVmax	9.90 ± 5.61	6.09 ± 2.84	<0.01
Recurrent tumors			
No. of lesions	18	18	
Mean SUVmax	9.44 ± 5.34	11.14 ± 4.80	0.28
Positive lymph nodes			
No. of lesions	137	108	
Mean SUVmax	7.95 ± 2.75	5.55 ± 1.59	0.01
Positive bone lesions			
No. of lesions	384	263	
Mean SUVmax	7.74 ± 3.72	5.66 ± 3.55	0.04
Positive liver lesions			
No. of lesions	55	48	
Mean SUVmax	5.13 ± 1.60	5.91 ± 3.29	0.60
Positive lung lesions			
No. of lesions	35	30	
Mean SUVmax	2.89 ± 1.23	3.37 ± 2.87	0.37
Positive adrenal lesions			
No. of lesions	4	4	
Mean SUVmax	3.43 ± 2.54	7.21 ± 4.60	0.10
Positive pleural lesions			
No. of lesions	59	25	
Mean SUVmax	4.54 ± 1.15	3.58 ± 2.40	0.53
Positive brain lesions			
No. of lesions	1	1	
Mean SUVmax	6.62	6.49	–

^68^Ga-FAP-2286 = Gallium 68 (^68^Ga) labeled fibroblast activating protein (FAP)-2286, ^18^F-FDG=Fluorine 18 (^18^F) labeled fluorodeoxyglucose (FDG), SUVmax=maximum standardized uptake value. P <0.05 indicates statistically significant differences.

### 3.3 Detection of lymph node metastasis

**Figure 2:**
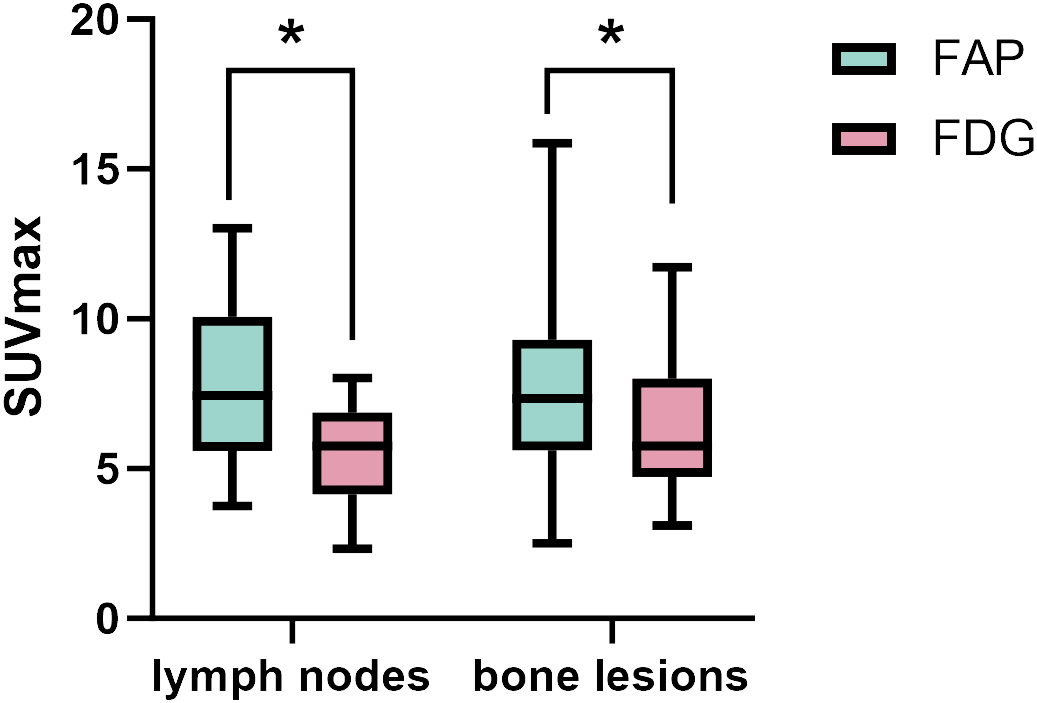
The SUVmax of FAP-2286 and FDG in lymph node and bone lesions. ^68^Ga-FAP-2286 accumulation was significantly higher than ^18^F-FDG accumulation in both lymph node metastasis (7.95 ± 2.75 vs.5.55 ± 1.59; P=0.01), and bone lesions (7.74 ± 3.72 vs. 5.66 ± 3.55, P=0.04). * indicates P < 0.05.

Among the 31 patients, 137 lymph node metastases were examined in 23 patients, and all 137 lymph nodes were accurately identified for lymph node involvement using ^68^Ga-FAP-2286 PET/CT, whereas only 108 of the 137 lymph node metastases were correctly diagnosed employing ^18^F-FDG PET/CT. The detection sensitivity of lymph nodes using ^68^Ga-FAP-2286 PET/CT was superior to that with ^18^F-FDG PET/CT [100% (137/137) vs. 78.8% (108/137), respectively]. Furthermore, the SUVmax value obtained from ^68^Ga-FAP-2286 PET was significantly higher than that from ^18^F-FDG PET for all the assessed nodal metastases (7.95 ± 2.75 vs. 5.55 ± 1.59, respectively, P=0.01, **Figure 2**). Notably, compared to ^18^F-FDG PET/CT, the detection efficacy of metastatic lymph nodes was notably improved with the utilization of ^68^Ga-FAP-2286 PET/CT.

### 3.4 Detection of bone and visceral metastasis

**Figure 3:**
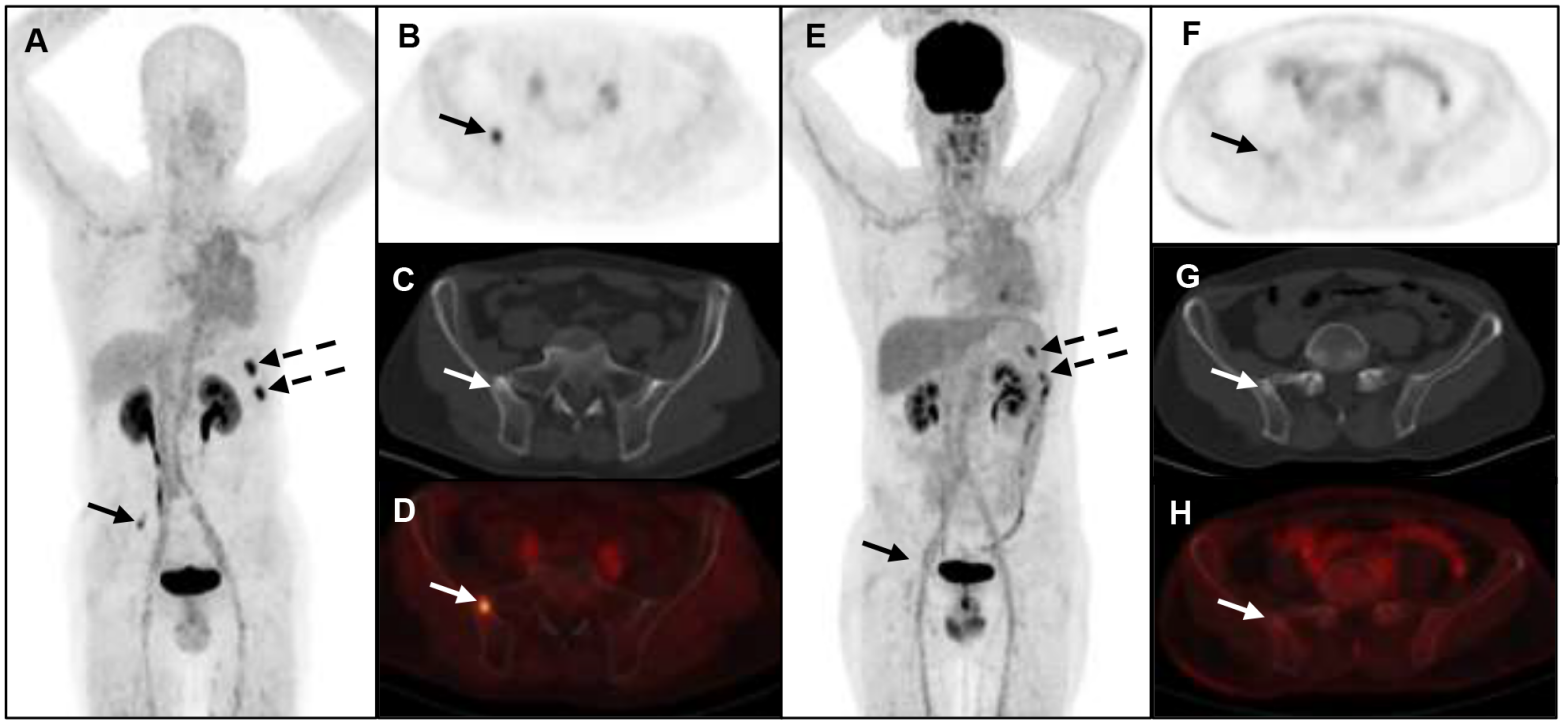
A 52-year-old man with metastatic lung cancer underwent cancer restaging imaging. ^68^Ga-FAP-2286 **(A-D)** uptakes in ilium (straight arrow, SUVmax 5.24) and left ribs (dotted arrow, SUVmax 8.12). ^18^F-FDG PET/CT **(E-H)** shows uptake in lower left ribs (dotted arrow, SUVmax 5.26), and no uptake in ilium.

**Figure 4:**
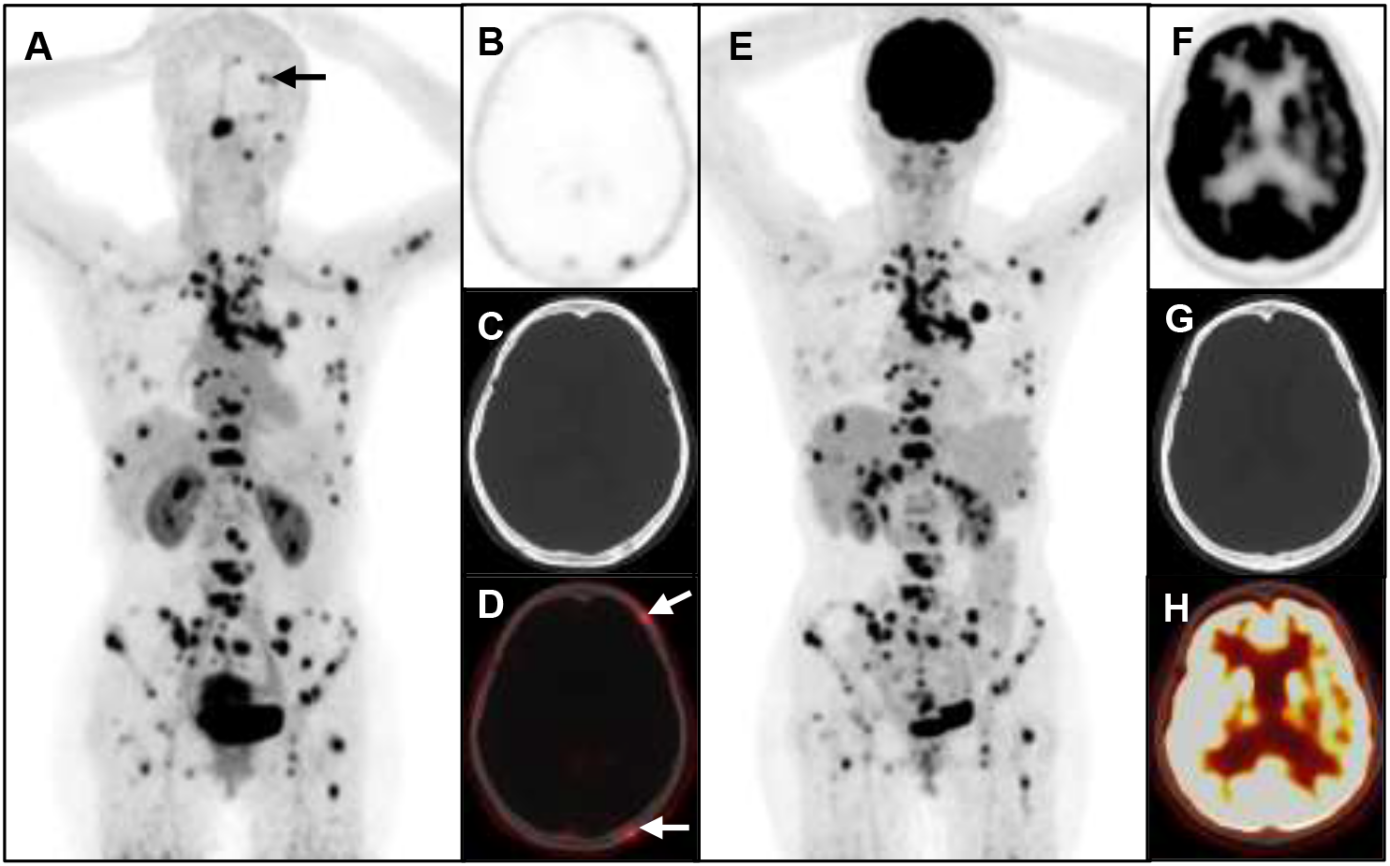
A 53-year-old woman with metastatic lung cancer underwent cancer restaging imaging. ^68^Ga-FAP-2286 shows higher uptake in skull lesions **(A-D)**, straight arrow, SUVmax 3.93).^18^F-FDG shows no uptake in skull lesions **(E-H)**.

A total of 6 different distant sites of involvement and 538 metastases were identified in 31 patients according to the gold and reference standards. ^68^Ga-FAP-2286 PET/CT detected all these lesions and 538 of 538 metastases were correctly identified. Meanwhile, ^18^F-FDG PET/CT detected all these lesions and 371 of 538 metastases. The SUVmax of bone metastases on ^68^Ga-FAP-2286 PET/CT images significantly differed from that on ^18^F-FDG PET/CT images (7.74 ± 3.72 vs. 5.66 ± 3.55, respectively, P=0.04, **Figure 2**). ^68^Ga-FAP-2286 PET/CT detected more bone lesions than ^18^F-FDG PET/CT [100% (384/384) vs. 68.5% (263/384), respectively] (**Table 2**, **Figure 3**), particularly in the skull [100% (14/14) vs. 14.3% (2/14), respectively] and rib [100% (72/72) vs. 27.5% (27/72), respectively] metastases (**Table 3**, **Figure 4**).

**Table 3:** Bone-positive lesions detected by ^18^F-FDG and ^68^Ga-FAP-2286 PET/CT.

Bone	^68^Ga-FAP-2286	Sensitivity	^18^F-FDG PET/CT	Sensitivity
**Skull**	14	100% (14/14)	2	14.3% (2/14)
**Scapula**	25	100% (25/25)	13	52% (13/25)
**vertebra**	152	100% (152/152)	126	82.4% (126/153)
**Ribs**	72	100% (72/72)	27	27.5% (27/72)
**Clavicle**	3	100% (3/3)	2	66.7% (2/3)
**Sternum**	15	100% (15/15)	9	60.0% (9/15)
**Pelvis**	72	100% (72/72)	63	87.5% (63/72)
**Long bones**	31	100% (31/31)	21	67.7% (21/31)
**All**	384	100% (384/384)	263	68.5% (263/384)

Numbers in parentheses are percentages. ^68^Ga-FAP-2286=Gallium 68 (^68^Ga) labeled fibroblast activating protein 2286; ^18^F-FDG=Fluorine 18 (^18^F) labeled fluorodeoxyglucose (FDG).

**Figure 5:**
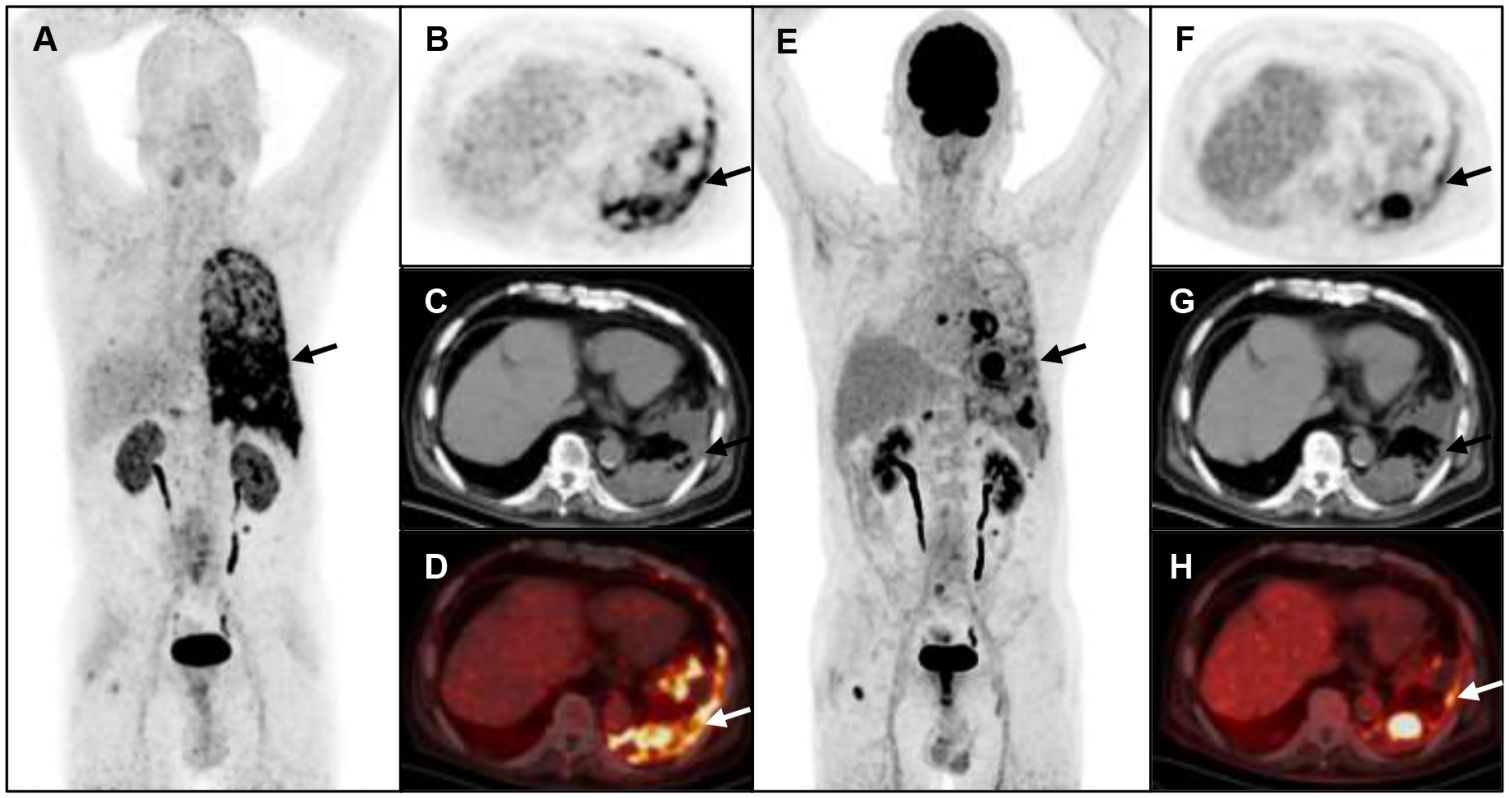
A 75-year-old man with metastatic lung cancer underwent cancer restaging imaging. ^68^Ga-FAP-2286 image shows more metastasis and pleural uptake **(A-D)**, straight arrow, SUVmax 9.83).^18^F-FDG shows lower uptake in pleural **(E-H)**, straight arrow, SUVmax 5.7).

Furthermore, pleural metastases were identified in four participants, with ^68^Ga-FAP-2286 PET/CT exhibiting superiority over ^18^F-FDG PET/CT in detecting metastatic lesions [100% (59/59) vs. 42.4% (25/59), respectively]. The SUVmax for positive pleural lesions of ^68^Ga-FAP-2286 PET/CT was not significantly different from that of 18F-FDG PET/CT (4.54 ± 1.15 vs. 3.58 ± 2.40, respectively, P=0.53) (**Table 2**, **Figure 5**).

For other visceral metastases, there was no significant difference in the detection rate between ^68^Ga-FAP-2286 and ^18^F-FDG for liver metastases [100% (55/55) vs. 87.2% (48/55), respectively] and lung metastases [100% (35/35) vs. 85.7% (30/35), respectively]. The SUVmax of ^68^Ga-FAP-2286 was not significantly different from that of ^18^F-FDG in liver lesions (5.13 ± 1.60 vs. 5.91 ± 3.29, respectively, P=0.60) and lung lesions (2.89 ± 1.23 vs. 3.37 ± 2.87, respectively, P=0.37).^68^Ga-FAP-2286 and ^18^F-FDG showed similar abilities to detect other suspected metastases, such as those in the adrenal glands and brain (**Table 2**).

## 4 Discussion

In this study, we used ^68^Ga-FAP-2286 PET/CT for patients with lung cancer and compared it with ^18^F-FDG. Our results showed that ^68^Ga-FAP-2286 PET/CT was superior to ^18^F-FDG PET/CT in detecting suspected lung cancer metastases to the lymph nodes, bone, and pleura.

Mediastinal lymph nodes serve as crucial predictors of lung cancer survival, and ^18^F-FDG PET/CT helps improve the accuracy of mediastinal staging. However, some limitations remain. In addition to the low sensitivity in detecting occult lymph node metastases (21), the heightened lymph node uptake observed in ^18^F-FDG PET/CT mandates caution against false positives. This can arise from inflammation, reactive hyperplasia, or infectious diseases such as post-stenosis pneumonia, particularly common in central tumors (22, 23). ^68^Ga-FAP-2286 reflects the activity of cancer-associated fibroblasts, which may also explain why the SUVmax in ^18^F-FDG PET/CT images was higher than in ^68^Ga-FAP-2286 PET/CT images (24). Additionally, it has been suggested that ^68^Ga-FAP-2286 is more suitable than ^18^F-FDG in distinguishing between reactive and metastatic lymph nodes (14). In our study, ^68^Ga-FAP-2286 PET/CT not only detected more positive lymph nodes than ^18^F-FDG PET/CT but also identified latent lesions that were easily overlooked because of very low or no uptake. Consequently, ^68^Ga-FAP-2286 PET/CT may help improve the accuracy of mediastinal staging and has the potential to detect early lung cancer.

In addition, ^68^Ga-FAP-2286 PET/CT exhibits a clear advantage in detecting bone metastases in lung cancer, revealing more suspicious bone metastases, especially bone lesions in the skull, ribs, and vertebrae (**Table 3**). Brain and bone metastases remain the leading causes of morbidity and mortality in patients with lung cancer (25, 26); hence, there is a need for early and accurate diagnosis of these diseases. Owing to the high physiological uptake rate of ^18^F-FDG in the brain, the sensitivity for the diagnosis of brain metastases is low (27), potentially impacting the assessment of skull metastases. In contrast, the accumulation of ^68^Ga-FAP-2286 on PET/CT in the brain tissue is minimal, ensuring that intracranial metastatic tumors are clearly detectable without interference from brain tissue uptake, particularly in the case of skull metastasis (28). Consequently, ^68^Ga-FAP-2286 PET/CT may offer a more advantageous diagnostic tool for brain and skull metastases than ^18^F-FDG PET/CT.

Our study also found that, due to pleural metastases exhibiting higher SUVmax uptake on ^68^Ga-FAP-2286 PET/CT, this imaging modality provided clear visualization of pleural metastases and identified more pleural lesions compared to ^18^F-FDG PET/CT. Furthermore, considering that pleural effusion is a common feature in patients with lung cancer (29), ^68^Ga-FAP-2286 PET/CT demonstrated the capability to detect additional concurrent pleural abnormalities. This capability contributes pertinent information to the assessment of suspected malignant pleural effusion and facilitates treatment decisions (30). Consequently, ^68^Ga-FAP-2286 PET/CT emerges as a new potential tool for evaluating malignant pleural lesions and warrants further study (31).

Notably, based on semi-quantitative analysis, the SUVmax of 18F-FDG PET/CT for distant metastatic lesions—such as lung cancer recurrence, intrapulmonary metastasis, and liver metastasis—was not significantly different from that observed in ^68^Ga-FAP-2286 PET/CT. However, it is important to acknowledge the potential bias introduced by the small sample size. Additionally, aside from the previously mentioned false-positive lesions that may occur in ^18^F-FDG PET/CT, we propose that this difference may also be linked to the heightened glucose metabolism in the liver, causing increased FDG uptake by intrahepatic metastatic lesions. However, the elevated FDG uptake in the liver background might obscure certain occult lesions. In contrast, FAP-2286 exhibits minimal uptake in normal tissues, contributing to improved tumor characterization.

Furthermore, this discrepancy could also be associated with the differentiation status of the tumor. Previous studies have suggested that highly and poorly differentiated liver cancers demonstrate relatively low or elevated FAPI uptake, respectively (32). This differentiation status detection principle may apply to other FAP-expressing tumors when utilizing FAP-2286.

However, histopathological results are unavailable for all highly suspected distant metastases, prompting questions regarding whether distant metastases positive in both tests should be unequivocally considered true metastases. Additionally, the inquiry arises as to whether the varying levels of uptake observed with different tracers can offer insights into the differentiation status of the tumor to some extent. Therefore, the difference in the uptake of ^18^F-FDG and ^68^Ga-FAP-2286 in distant metastatic lesions and its mechanism need to be further verified by histopathological results. At the same time, image interpretation should consider other imaging findings and clinical data rather than solely relying on the level of tracer uptake.

In comparison to the small molecule FAPI family (FAPI-04/46/74), FAP-2286 incorporates cyclic peptides as binding motifs, offering potential improvements in biological properties such as enhanced receptor selectivity and binding affinity due to increased plasma stability and conformational rigidity (33). In a preclinical model, ^68^Ga-FAP-2286 showed high tumor uptake and retention (13), which may help diagnose solid tumors, especially malignancies with low to moderate uptake on ^18^F-FDG PET/CT, including lung, stomach, pancreatic, and liver cancers (14). It also has unique advantages in diagnosing lung cancer and brain metastases. Pang et al. ‘s findings also indicate that ^68^Ga-FAP-2286 is a promising FAPI molecule for safe cancer diagnosis, staging, and re-staging (14).

Moreover, preclinical studies and initial human trials indicate the efficacy of ^177^Lu-FAP-2286 against FAP-positive tumors (13, 34). Studies on its biological distribution in mice showed that ^177^Lu-FAP-2286 was rapidly and consistently taken up by FAP-positive tumors, cleared by the kidneys, and taken up very little in normal tissues without significant weight loss (13). Additionally, ^177^Lu-FAP-2286 (5.8 ± 2.0 GBq; range, 2.4–9.9 GBq) was well tolerated, had significant tumor uptake and long-term retention, and showed a similar biological distribution on SPECT/CT as the imaging agent ^68^Ga-FAP-2286. No adverse symptoms or clinically detectable pharmacological effects were found or reported in participants, and it alleviated pain symptoms in three patients with advanced disease (34). Preliminary results from the Phase 1/2 clinical trial (NCT04939610) also reported that ^177^Lu-FAP-2286 showed a manageable safety profile in nine patients with seven different cancers (35). Recent studies have also shown that ^177^Lu-FAP-2286, when combined with PD-1 checkpoint inhibition, can enhance tumor efficacy (36). Based on the results of this study, the benign imaging of ^68^Ga-FAP-2286 in advanced lung cancer, especially the imaging of metastatic lymph nodes and bone lesions, provides a basis for the treatment of ^177^Lu-FAP-2286 in metastatic lung cancer.

Overall, ^68^Ga-FAP-2286 PET provides valuable information for clinical management, including the identification of false-negative results from routine imaging and false-positive results from ^18^F-FDG PET (17). Improvements in tumor detectability may lead to changes in clinical staging and optimization of treatment strategies (37). Moreover, FAP-2286 has shown higher SUV_max_ values than FDG in many studies, and a good tumor-to-background ratio may improve the delineation of gross tumors in radiotherapy and the evaluation of therapeutic efficacy (16, 18, 37, 38). This new imaging approach can also inform clinical decision-making by improving local lymph node staging and identifying metastatic disease that cannot be detected by conventional imaging. This is critical for determining patient treatment options and prognosis (17). Because of these advantages, FAP-2286 is considered a potential compound for treating patients with advanced metastatic cancer (16).

In a first-in-human trial in 11 patients, FAP-2286 demonstrated its utility as a therapeutic agent, with labeled ^68^Ga used for diagnosis and ^177^Lu for therapy (30). Recent case reports have also demonstrated the remarkable efficacy of ^177^Lu-FAP-2286 against different types of epithelial cancer, offering a new treatment option to control disease progression and improve patient survival (39–42). However, further large-scale prospective studies on ^68^Ga/^177^Lu-FAP-2286 are needed to evaluate its safety, pharmacokinetics, dosimetry, and efficacy for patient management and integration.

This study has some limitations. First, the diagnoses of lymph nodes and distant metastases were primarily determined by imaging, and not all highly suspected metastatic lesions had histopathological confirmation. Second, we did not measure the target background ratio, and the description of the imaging diagnosis was not sufficiently comprehensive. Third, most patients in our study had stage IV lung cancer, which does not reflect the full spectrum of ^68^Ga-FAP-2286 PET/CT findings in lung cancer. Finally, the small sample size and the large gender distribution gap of enrolled patients might have led to bias in the overall study results. In the future, multicenter clinical studies will be conducted to include a larger number of lung cancer at different stages to overcome these limitations.

## 5 Conclusion

Compared to ^18^F-FDG PET/CT, ^68^Ga-FAP-2286 PET/CT demonstrated better lesion detection capabilities for lung cancer, particularly in lymph nodes and bone metastases, providing compelling imaging evidence for the efficacy of ^177^Lu-FAP-2286 treatment.

## Data availability statement

The original contributions presented in the study are included in the article/supplementary material, further inquiries can be directed to the corresponding authors.

## Ethics statement

The study was approved by the Affiliated Hospital of Southwest Medical Universityl Review Board, registered on the Chinese Clinical Trial Registry website (http://chictr.org.cn, ChiCTR2100044131), and obtained ethical clearance from the Ethics Committee (AHSWMU-2020-035). The studies were conducted in accordance with the local legislation and institutional requirements. All participants provided written informed consent before receiving treatment. Written informed consent was obtained from the individual(s) for the publication of any potentially identifiable images or data included in this article.

## Author contributions

FX: Conceptualization, Data curation, Visualization, Writing – original draft, Writing – review & editing. YZ: Data curation, Formal Analysis, Software, Writing – original draft. XT: Methodology, Software, Writing – review & editing. JZ: Formal Analysis, Resources, Writing – review & editing. TL: Supervision, Validation, Writing – review & editing. YY: Formal Analysis, Investigation, Writing – review & editing. WM: Project administration, Supervision, Validation, Writing – review & editing. YC: Funding acquisition, Project administration, Resources, Writing – review & editing.
